# Differential physiological and production responses of C3 and C4 crops to climate factor interactions

**DOI:** 10.3389/fpls.2024.1345462

**Published:** 2024-02-02

**Authors:** Emmanuel Opoku, Pranav Pankaj Sahu, Hana Findurová, Petr Holub, Otmar Urban, Karel Klem

**Affiliations:** ^1^ Laboratory of Ecological Plant Physiology, Global Change Research Institute Czech Academy of Sciences (CAS), Brno, Czechia; ^2^ Department of Agrosystems and Bioclimatology, Faculty of AgriSciences, Mendel University in Brno, Brno, Czechia

**Keywords:** photosynthetic metabolism, *Hordeum vulgare*, *Sorghum bicolor*, biomass production, physiology, warming, elevated CO_2_ concentration, water stress

## Abstract

This study examined the effect of the interactions of key factors associated with predicted climate change (increased temperature, and drought) and elevated CO_2_ concentration on C3 and C4 crop representatives, barley and sorghum. The effect of two levels of atmospheric CO_2_ concentration (400 and 800 ppm), three levels of temperature regime (21/7, 26/12 and 33/19°C) and two regimes of water availability (simulation of drought by gradual reduction of irrigation and well-watered control) in all combinations was investigated in a pot experiment within growth chambers for barley variety Bojos and sorghum variety Ruby. Due to differences in photosynthetic metabolism in C3 barley and C4 sorghum, leading to different responses to elevated CO_2_ concentration, we hypothesized mitigation of the negative drought impact in barley under elevated CO_2_ concentration and, conversely, improved performance of sorghum at high temperatures. The results demonstrate the decoupling of photosynthetic CO_2_ assimilation and production parameters in sorghum. High temperatures and elevated CO_2_ concentration resulted in a significant increase in sorghum above- and below-ground biomass under sufficient water availability despite the enhanced sensitivity of photosynthesis to high temperatures. However, the negative effect of drought is amplified by the effect of high temperature, similarly for biomass and photosynthetic rates. Sorghum also showed a mitigating effect of elevated CO_2_ concentration on the negative drought impact, particularly in reducing the decrease of relative water content in leaves. In barley, no significant factor interactions were observed, indicating the absence of mitigating the negative drought effects by elevated CO_2_ concentration. These complex interactions imply that, unlike barley, sorghum can be predicted to have a much higher variability in response to climate change. However, under conditions combining elevated CO_2_ concentration, high temperature, and sufficient water availability, the outperforming of C4 crops can be expected. On the contrary, the C3 crops can be expected to perform even better under drought conditions when accompanied by lower temperatures.

## 1 Introduction

The rising atmospheric concentrations of greenhouse gases, especially CO_2_, have led in recent decades to rising air temperatures and the increasing frequency of climate extremes (Sage, 2020; Zandalinas et al., 2021). Extreme climate events including temperature extremes and anomalies in intensity, duration and frequency of precipitation in recent years have significantly affected crop growth and yield (Fahad et al., 2017). Such environmental changes, together with the decline in arable land, are expected to impact crop production and subsequently lead to global food insecurity (Borrelli et al., 2020; Sage, 2020; Zandalinas et al., 2021), although global food demand is projected to rise (Conijn et al., 2018).

Cereals are a critical component of the world’s agricultural industry, providing a significant source of food for mankind and feed for livestock (McKenzie and Williams, 2015; Bruinsma, 2017). The varying responses of different crop species and varieties to environmental stress have been shown to be influenced by their genetic backgrounds. There are a number of known regulation or resistance mechanisms that contribute significantly to crop resilience to adverse environmental conditions, as well as a number of genomics or phenomics tools that can be effectively used for crop improvement (Raza et al., 2019). However, the change in the crop structure specific for the given conditions, in particular a change in the proportion of C3 and C4 crops, together with the adaptation of crop management, will also be a vital tool for adaptation of crop production to climate change (Rezaei et al., 2023). The performance and the applicability of different resistance mechanisms are at this point relatively well explored for individual stressors. However, there are still many gaps in our understanding of their significance when multiple environmental factors act simultaneously, with responses ranging from synergism to antagonism (Zandalinas and Mittler, 2022), although crop modelling provides effective ways to estimate future impacts of climate change based on combining several factors and to evaluate potential differences in responses between C3 and C4 crops (Wang et al., 2023).

Drought stress can decrease photosynthesis, and nutrient uptake in plants, leading to a decrease in biomass production and grain yield. Plants respond to drought stress by activating a series of physiological and molecular mechanisms, including the closure of stomata, the accumulation of osmolytes and antioxidants, and changes in root architecture (Farooq et al., 2009; Anjum et al., 2011; Zhao et al., 2020).

Elevated atmospheric CO_2_ concentrations can enhance photosynthesis and water use efficiency in plants, resulting in higher biomass production and yield under favourable growing conditions (Leakey et al., 2019; Souza et al., 2019) and mitigating negative effects of reduced water availability (Abdelhakim et al., 2022). However, an increase in leaf area index under elevated CO_2_ concentration and thus increased transpiration per unit ground area may completely counteract its mitigating effect or even lead to an amplification of the drought effect (Burkart et al., 2011).

High temperature is another critical abiotic stress that can significantly impact cereal growth and yield, particularly during the reproductive stage. High temperatures can affect various physiological and biochemical processes in plants, including photosynthesis, respiration, and stress signalling pathways (Tiwari and Yadav, 2019). Plants respond to high temperatures by activating a series of protective mechanisms, including the synthesis of heat shock proteins, the accumulation of antioxidants, and changes in membrane fluidity (Jat et al., 2016). However, prolonged exposure to high temperatures can lead to irreversible damage to plant tissues and even death.

Understanding the physiological and morphological responses of cereals to drought stress, elevated CO_2_, and high temperature is therefore essential for development of adaptation strategies including crop selection, development of climate-resilient crop varieties and sustainable agricultural practices, that can help to cope with the changing climate. In comparison to C4, C3 crops have a reduced capacity to withstand short-term drought stress due to lower water use efficiency (Hura et al., 2007), but they exhibit a more positive response to the elevated CO_2_ concentration due to reduced photorespiration and enhanced photosynthetic CO_2_ uptake (Drake et al., 1997). Due to the higher response of C3 crops to elevated CO_2_ concentration, its effect is also manifested in the mitigation of drought stress, either by increasing water use efficiency or by its positive effect on photosynthesis and yield (van der Kooi et al., 2016; Abdelhakim et al., 2022). In addition, C4 crops show a lower threshold of decrease in leaf water potential from which a decrease in photosynthesis is observed compared to C3 crops and also a higher sensitivity of non-photochemical limitation of photosynthesis to drought (Bellasio et al., 2023). Thus, despite the higher water use efficiency, higher drought sensitivity is generally observed in C4 plants, with elevated CO_2_ concentrations exacerbating these differences between C4 and C3 plants (van der Kooi et al., 2016). Elevated CO_2_ concentration in combination with adaptation measures is expected to compensate yield losses in C3 crops caused by drought, while the possibility of compensating for yield losses is limited for C4 crops (Makowski et al., 2020). Even more complex is the assessment of the effect of high temperature in C3 and C4 crops and, in particular, the interactions with elevated CO_2_ concentration and drought. Despite recent studies have attempted to understand the different interactions of environmental conditions associated with climate change, either through experiments or the use of crop models, it is still very difficult to generalise under which combinations of factors C3 crops will outperform C4 crops and vice versa (Rakhmankulova et al., 2023; Wang et al., 2023). Although C4 plants show a higher temperature optimum for photosynthesis (Yamori et al., 2014), a higher sensitivity to acute heat stress is observed, which is further amplified by the combination with elevated CO_2_ concentration (Hamilton et al., 2008). The complex physiological and biochemical background of these interactions, which is not yet fully understood, leads to rather contradictory results of the interactions of elevated CO_2_ concentration, high temperature and drought in C3 and C4 crops and thus to highly uncertain yield predictions in the context of expected climate change.

In this study, we aimed to investigate differences between C4 (sorghum) and C3 (barley) crop representatives in the interactive effects of elevated CO_2_ concentration, high temperature and drought on physiological and production parameters. Our hypotheses were as follows: i) C4 sorghum exhibits higher drought tolerance due to higher water use efficiency and also greater tolerance to high temperatures. ii) C3 barley exhibits a higher mitigating effect of elevated CO_2_ concentration against drought due to greater stomatal response and stimulation of photosynthesis. iii) Elevated CO_2_ concentration therefore compensates for differences between C3 and C4 crops in response to drought, but does not affect differences in response to high temperatures.

## 2 Materials and methods

### 2.1 Plant growth and experimental regimes

**Figure 1:**
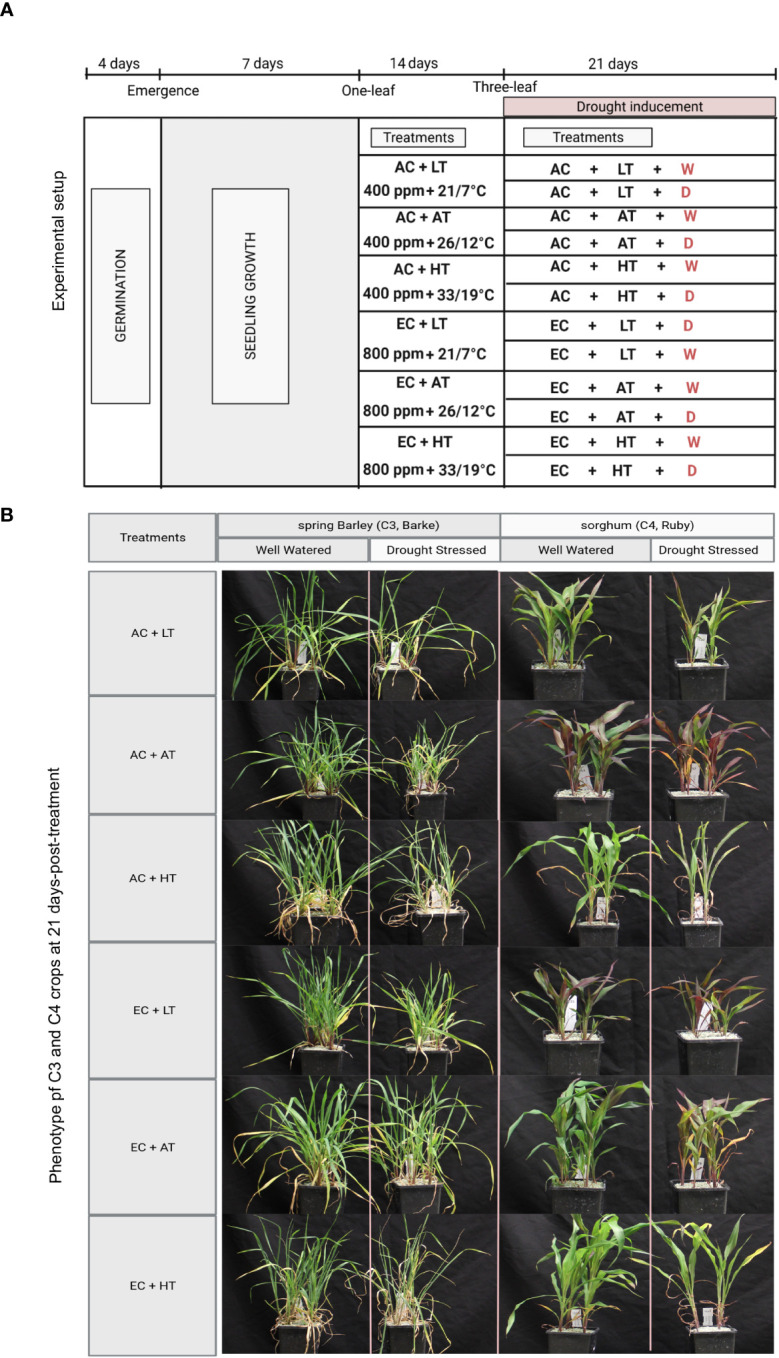
Experimental setup and crop phenotypes after exposition to individual treatments. **(A)** The figure represents the time course of manipulation of individual environmental factors. Seedlings from both C3 (barley, variety Barke) and C4 (sorghum, variety Ruby) crop species were subjected to seed germination (4 days) and early seedling growth (7 days) before starting the CO_2_ concentration and temperature treatments. The seedlings (one leaf stage) were then exposed to 6 different combinatorial treatments of CO_2_ concentration (AC – 400 ppm and EC – 800 ppm) and temperature (LT – 21/7°C, AT – 26/12°C and HT – 33/19°C). After 14-day acclimation, progressive drought stress was applied by reducing the watering gradually **(D)**, except for the well water conditions (W). **(B)** The phenotype of barley and sorghum after 21 days exposition to drought in combination with CO_2_ and temperature treatment.

The study was conducted on two genotypes that represented C3 species spring barley (*Hordeum vulgare*, variety Barke) and C4 species sorghum (*Sorghum bicolor*, variety Ruby), respectively. In September 2022, four germinating seeds were planted in each plastic pot (11 × 11 × 25 cm) after stratification for 48 hours at 4°C and germination for 48 hours at 26°C on moistened filter paper. In total 120 pots were filled with Zeolite with granulation of 2.5-5 mm (Techneco, Praha, Czech Republic) after inserting the 10 mm thick Grodan plate on the pot bottom. This ensured a gradual drainage of excess watering and at the same time prevented the Zeolite granules from falling out through the drainage holes of the pot. The pots were left in the greenhouse at ambient temperature (20 ± 5°C) and regularly irrigated until seedling started to emerge. The pots were then transferred to six growth chambers model FytoScope FS-SI 3400 (PSI, Drásov, Czech Republic). Seedlings of both species were initially allowed to acclimate for seven days (before the respective treatments) under identical environmental conditions, simulating the changes during daily course: air temperature (day maximum/night minimum, 26/12°C), relative air humidity (day minimum/night maximum, 70/90%), photosynthetically active radiation intensity (day maximum/night, 800/0 μmol photons m^-2^ s^-1^), constant CO_2_ concentration (400 ppm), and day/night duration 15/9 h (**Figure 1A**). All pots were irrigated to keep Zeolite at full water holding capacity every 2 days. Afterwards, when the plants reached the stage of one fully developed leaf (7 days after starting germination), they were exposed to the experimental treatments. Each combination of species, CO_2_ concentration, temperature regime, and water availability were replicated five times (five pots), and the replications were randomized within the blocs at an interval of 3-4 days to minimise the effects of possible inhomogeneity of environmental conditions inside the chambers. To avoid the possible artefacts of individual chambers, treatments were interchanged between chambers at an interval of one week. The experimental treatments included three temperature regimes comprising of low temperature (LT), ambient temperature (AT), and high temperature (HT) with day maximum/night minimum air temperatures of 21/7°C, 26/12°C and 33/19°C, respectively (**Figure 1A**). The temperature regimes selected simulate a below-average cool season (LT), a normal season (AT) and an above-average warm season (HT). HT regime demonstrates not only the furure warming but also a higher frequency of episodes of high temperatures (HT), under Central European conditions for the stem elongation period of both crops (May-June). Atmospheric CO_2_ concentration was maintained at a constant level ( ± 50 ppm) in two regimes including ambient CO_2_ concentration (AC, 400 ppm) and elevated CO_2_ concentration (EC, 800 ppm). The CO_2_ concentration of 800 ppm represents an estimate of the change in atmospheric CO_2_ concentration by 2100 under the SRES A2 (business as usual) emissions scenario (Valone, 2021). Relative air humidity for all treatments was identical with a day minimum/night maximum of 60/90%.

Plants were watered using Knop´s nutrient solution every two days. The nutrient solution in distilled water contained the following concentrations of compounds: Ca (NO_3_)_2_*4H_2_O 1.439 g L^-1^, MgSO_4_ 0.122 g L^-1^, KH_2_PO_4_ 0.25 g L^-1^, KCl 0.125 g L^-1^, and FeCl_3_ 0.006 g L^-1^. The resulting solution had a pH between 5 and 6. At the three-leaf stage (two weeks after induction of CO_2_ and temperature treatments), gradual drought induction was started. Drought (D) was initiated by reducing the amount of irrigation to 50% (7 days), followed by a reduction to 33% (10 days), and finally, irrigation was completely withdrawn (4 days), while the well-watered (W) plants were kept irrigated every two days with 80 ml of nutrient solution per pot. At the end of drought treatment (21 days after drought initiation), measurements of physiological parameters associated with photosynthetic CO_2_ assimilation, indirect measurement of leaf pigments, determination of above-ground and below-ground biomass per plant as well as sampling for elemental analyses were performed.

### 2.2 Leaf relative water content

Leaf relative water content (RWC) was determined after 21 days of drought treatment as described by (González and González-Vilar, 2001). The following equation was used to calculate the RWC:


$$\text{RWC}=\frac{\text{Fresh weight}-\text{dry weight}}{\text{Turgid weight }-\text{ dry weight}}\;\times 100$$

The measurement of fresh leaf segment weight was followed by the determination of turgid weight after the leaves were immersed in distilled water and placed for 12 hours in the dark at 4°C (Fletcher et al., 1988). The dry weight of leaf segments was then determined by a drying process at 70°C until a consistent weight was achieved.

### 2.3 Gas exchange measurements

Gas exchange measurements were conducted between 10:00 and 14:00 Central European Time (CET). Basic photosynthetic parameters were measured on the 2nd leaf from the top (youngest fully developed leaf), representing the leaf completely developed during drought induction, using a LI-6800 gas exchange system (LI-COR Biosciences, Lincoln, Nebraska, USA). The CO_2_ concentration in the leaf chamber was kept constant at a level of 400 and 800 ppm for AC and EC treatments, respectively. During gas-exchange measurements, air temperature and relative air humidity were kept constant in the growth chamber corresponding to daily maxima of 21°C/60%, 26°C/60% and 33°C/60% for LT, AT and HT, respectively. These conditions were also set up in the leaf chamber of the gas exchange analyser. To measure light-saturated CO_2_ assimilation rate (*A*_max_), the leaves were exposed to the photosynthetically active radiation intensity of 1200 μmol photons m^-2^ s^-1^. In parallel with *A*_max_, stomatal conductance (*G*_S_) and transpiration rate (*E*) were determined.

### 2.4 Leaf pigments and chlorophyll fluorescence parameters

Contents of leaf chlorophylls, epidermal flavonols and anthocyanins were estimated indirectly as chlorophyll (Chl), flavonol (Flav) and anthocyanin indices (Anth) using Dualex 4 Scientific (Force A, Orsay, France).

The actual quantum yield of photosystem II (Φ_PSII_) was determined using the Open FlourCam FC 800-O/2020 (PSI) after exposure to actinic light (150 μmol photons m^-2^ s^-1^) for 150 s. A saturating pulse (~2700 μmol photons m^-2^ s^-1^) was applied at the end of actinic light exposure to determine the maximum fluorescence (*F*_M_´) after the measurement of steady-state chlorophyll fluorescence under actinic light (*F*_S_). The actual quantum yield of photosystem II was calculated as follows: Φ_PSII_ = (*F*_M_´ - *F*_S_)/*F*_M_´.

### 2.5 Carbon and nitrogen content

Fully developed leaves, second from the top of barley and sorghum plants, were sampled at the end of drought treatment and then dried at 70°C to constant weight. Subsequently, dried leaves were homogenised using the MM400 mill (Retsch, Haan, Germany). Approximately 1.5 mg of the pulverised samples were weighted into tin capsules to determine leaf carbon and nitrogen contents using an elemental analyser Flash 2000 (ThermoScientific, Waltham, Massachusetts, USA).

### 2.6 Above- and below-ground biomass

Two plants per pot were used to determine above- and below-ground biomass. Plants were separated from the zeolite, the roots were gently washed on a fine sieve of mesh 0.3 mm under running water, the roots and above-ground biomass were separated, and then dried at 70°C to constant weight. The average of two plants from each pot (replicate) was then used for further statistical analyses.

### 2.7 Statistical analyses

Prior to conducting the analysis of variance (ANOVA), the Kolgomorov–Smirnov test was employed to assess the normality of data for each individual parameter. Parameters that failed the normality test were transformed using square root transformation. A four-way analysis of variance (ANOVA) was performed using the STATISTICA 12 (StatSoft, Tulsa, Oklahoma, USA) to determine main effects and interactions. Tukey’s *post-hoc* test (*p* = 0.05) following three-way ANOVA (separately for each species) was used to analyse significant differences between means. Pearson’s correlation matrix with *p*-values was used to create a correlation matrix by R package, *ggplot2* (Wickham, 2016). The square root normalized data of morpho-physiological traits were used to create heatmap by *Pheatmap* package in R. Boxplot graphs and PCA biplot were developed in the software OriginPro 2021b (OriginLab, Northampton, Massachusetts, USA).

## 3 Results

### 3.1 Associations between experimental factors and biomass, physiological and biochemical parameters

Our study was performed to understand the influence of different climate variables and their interactions on C3 and C4 crop species. All twelve treatments represent the combinations of three environmental factors atmospheric CO_2_ concentration, temperature, and water availability while the relative air humidity and photosynthetically active radiation intensity were not modified (**Figure 1A**).

**Figure 2:**
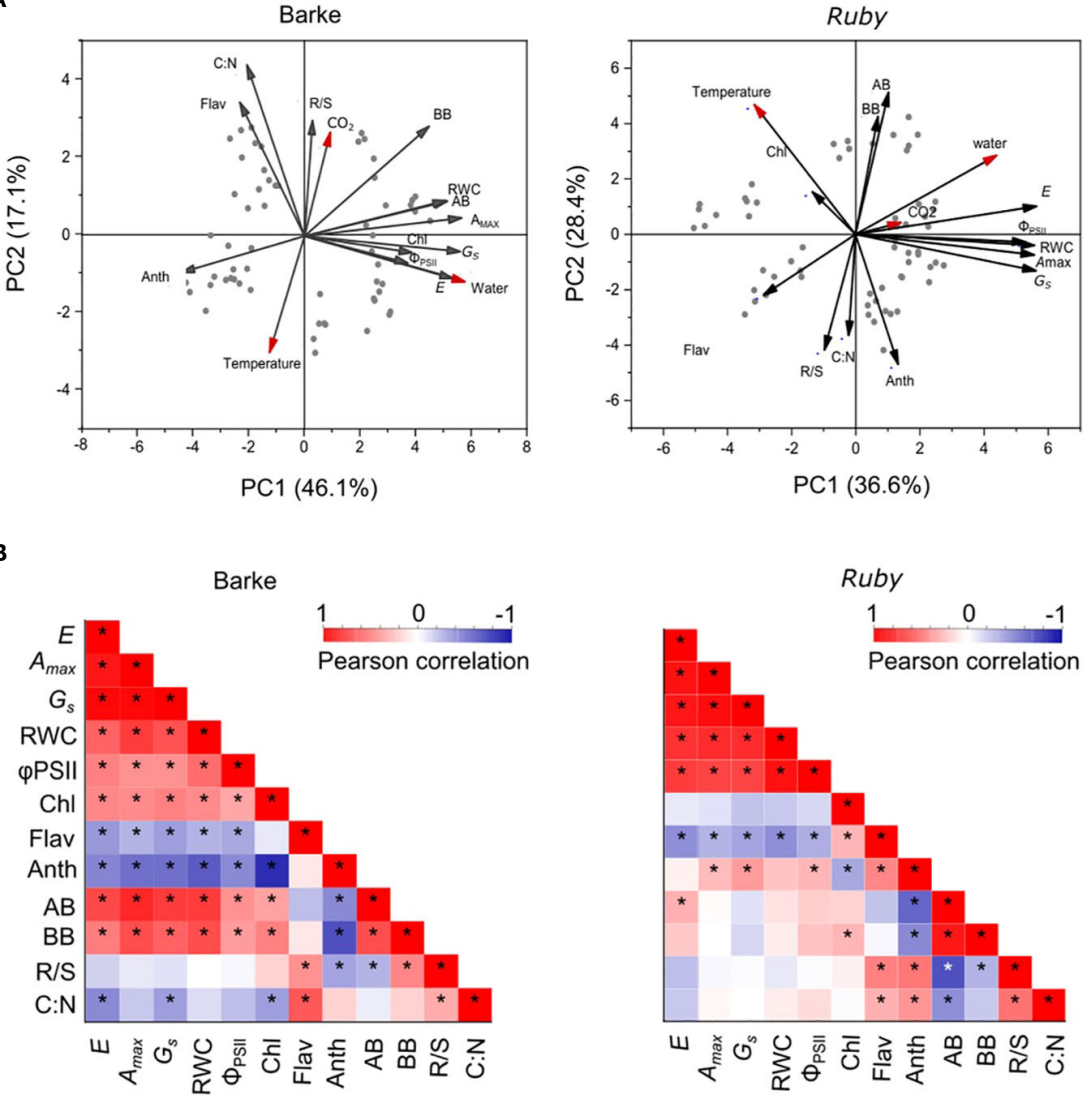
PCA and correlation analysis of physiological, production and biochemical parameters in C3 (barley, Barke) and C4 (sorghum, Ruby) crops. **(A)** PCA analysis was performed separately for barley (variety Barke – left) and sorghum (variety Ruby – right). The red arrows represent the effect of experimental factors: CO_2_ – atmospheric CO_2_ concentration, Temperature –air temperature, Water – water availability. **(B)** The correlation matrices for each species separately, representing the strength, direction and significance of the relationships between each pair of measured production, physiological and biochemical parameters. The red colour represents a positive relationship while the blue colour represents a negative relationship. The intensity of the colour demonstrates the strength of the relationship (significant relationships are marked with an asterisk*). *A*_max_, light-saturated CO_2_ assimilation rate; *G*_S_, stomatal conductance; *E*, transpiration rate; Φ_PSII_, actual quantum yield of photosystem II; RWC, relative water content; Chl, chlorophyll index; Flav, flavonol index; Anth, anthocyanin index; AB, above-ground dry biomass per plant; BB, below-ground dry biomass per plant; R/S, root-to-shoot ratio (BB/AB); C:N, carbon-to-nitrogen ratio in plant dry matter.

PCA analysis performed separately for barley and sorghum shows some general species-specific features in response to elevated CO_2_ concentration, temperature, and water availability (**Figure 2A**). The main differences are in the response to higher temperatures and elevated CO_2_ concentration. While barley shows a negative association between below-ground biomass or root-to-shoot ratio (R/S) to temperature, sorghum exhibits a rather positive association with temperature for both above- and below-ground biomass (**Figure 2A**). The changes in R/S in sorghum are rather related to the combined effect of temperature and water availability (higher temperature and increased water availability negatively affect R/S). In barley, R/S is positively associated with elevated CO_2_ concentration, but negatively with high temperature. Above-ground biomass in barley is also positively associated with most physiological parameters, which are primarily affected by water availability, whereas this association is low in sorghum. Although physiological parameters are also largely influenced by water availability, the interaction of temperature and water availability has a major effect on production parameters in sorghum. In sorghum, the effect of elevated CO_2_ concentration is significantly lower. R/S is also positively associated with the C:N ratio in above-ground biomass in both species (**Figure 2A**). In barley, Anth is then negatively associated with physiological parameters and positively with C:N ratio, while in sorghum a similar association is observed for Flav. This indicates that the role of flavonols and anthocyanins is somewhat reversed in barley and sorghum. Chl was relatively little affected by experimental factors and had a positive association with physiological parameters in barley, whereas in sorghum these associations were rather negative.

The correlation matrix evaluates the magnitude and direction of the relationship for each of the pair of measured parameters separately for barley and sorghum (**Figure 2B**). Both species show a highly positive correlation among the physiological parameters. Significant differences between the two species are apparent for the correlations of Chl. While the correlation of Chl to physiological parameters is positive and significant in barley, it is negative and insignificant in sorghum (**Figure 2B**). Chl is further negatively correlated with Anth in both species (more in barley), while Flav shows a significant positive correlation to Chl only in sorghum. The production parameters (above- and below-ground biomass) show a clear positive correlation to physiological parameters in barley, while these correlations are low, and in most cases insignificant in sorghum (**Figure 2B**). In contrast, the negative correlations of biomass parameters to Anth are very similar for both species. R/S and C:N correlate significantly positively in both species with Flav and, in the case of sorghum, also with Anth.

### 3.2 General effect of species, elevated CO_2_ concentration, temperature and water availability on production, physiological and biochemical parameters

**Figure 3:**
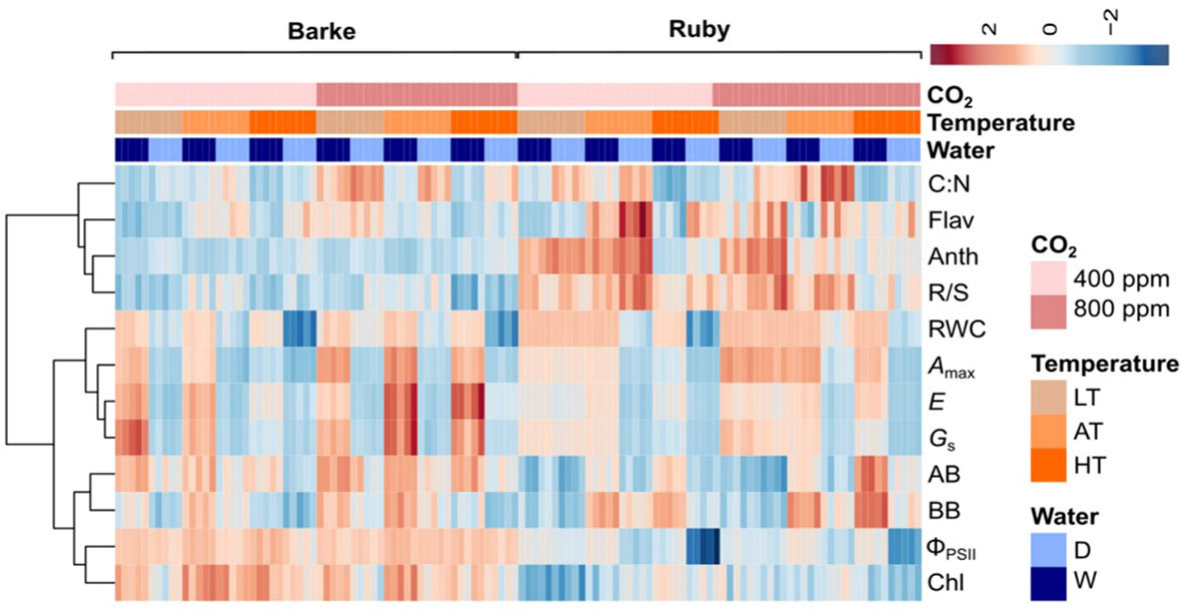
The heat map showing the effects of species, atmospheric CO_2_ concentration (400 ppm – AC, 800 ppm – EC), temperature (LT – 21/7°C, AT – 26/12°C and HT – 33/19°C) and water availability (D, drought stress; W, well-watered control). *A*_max_, light-saturated CO_2_ assimilation rate; *E*, transpiration rate; *G*_S_, stomatal conductance; Φ_PSII_, actual quantum yield of photosystem II; RWC, relative water content; Chl, chlorophyll index; Flav, flavonol index; Anth, anthocyanin index; AB, above-ground dry biomass per plant; BB, below-ground dry biomass per plant; R/S, root-to-shoot ratio (BB/AB); C: N, C:N ratio in plant dry matter.

The heat map shows that the predominant effect of species is particularly evident in the pigment content, i.e. Chl, which is higher in barley, Flav and Anth, which are higher in sorghum (**Figure 3**). Species-specific differences were also high in the case of the R/S ratio which is higher in sorghum and the fluorescence parameter Φ_PSII_ which is higher in barley. In the case of the other parameters, the effect of experimental factors is more significant and dominates over the effect of species. Atmospheric CO_2_ concentration shows a positive effect on most physiological parameters in sorghum, particularly in LT and AT, while in barley these parameters are predominately influenced by water availability (drought reduces physiological parameters) which is not evident or less pronounced in sorghum. The negative drought effect on above- and below-ground biomass in barley was more pronounced under AC as compared to EC and in HT, while in sorghum the drought effect dominated in HT irrespective of CO_2_ concentration.

A four-way ANOVA of the effect of experimental factors on plant physiological parameters, biomass production, N content, and C:N ratio in plant dry matter (**Table 1**) showed a statistically highly significant effect of species (*p* ≤ 0.01) for most parameters, except C:N ratio where the effect was insignificant. CO_2_ concentration showed a highly significant effect (*p* ≤ 0.01) on RWC, gas exchange parameters (*A*_max_, *G*_S_, *E*), Φ_PSII_, Anth, above- and below-ground biomass, and C:N ratio. In contrast, the effect of temperature was highly significant (*p* ≤ 0.01) for all measured biomass, physiological and biochemical parameters except for *E*. Similarly, drought had a highly significant statistical effect (*p* ≤ 0.01) on all measured parameters. Species and CO_2_ concentration showed highly significant interaction (*p* ≤ 0.01) on *E*, Chl, Anth and C:N ratio. The interaction between species and temperature was highly significant (*p* ≤ 0.01) for all observed parameters except for R/S. The interaction between CO_2_ concentration and the temperature was highly significant (*p* ≤ 0.01) for all observed parameters except for below-ground biomass where the interaction was insignificant and C:N where it was significant at the *p* ≤ 0.05 level. The interaction of species with drought was statistically highly significant for the parameters RWC, *A*_max_, *G*_S_, *E*, Φ_PSII_, Chl, Flav and below-ground biomass. The interaction of CO_2_ concentration and drought was highly statistically significant for the parameters RWC, *A*_max_, *G*_S_, *E* and Φ_PSII_ and significant at the *p* ≤ 0.05 level for the C:N ratio. The interaction between temperature and drought was highly significant for the parameters RWC, *A*_max_, *G*_S_, *E*, Φ_PSII_, above- and below-ground biomass. A significant interaction at the *p* ≤ 0.05 level was found for Flav.

**Table 1:** Results of a four-way analysis of variance (ANOVA) for the effect of each experimental factor separately and their mutual interactions.

Parameters	Species [S]	CO_2_	Temperature [T]	Water availability [WA]	S x CO_2_	S x T	CO_2_ x T	S x WA	CO_2_ x WA	T x WA
RWC	**<0.001**	**<0.001**	**<0.001**	**<0.001**	0.274	**<0.001**	**<0.001**	**<0.001**	**<0.001**	**<0.001**
*A*_max_	**<0.001**	**<0.001**	**<0.001**	**<0.001**	0.223	**<0.001**	**0.009**	**<0.001**	**<0.001**	**<0.001**
*G*_S_	**<0.001**	**<0.001**	**<0.001**	**<0.001**	0.026	**<0.001**	**<0.001**	**<0.001**	**<0.001**	**<0.001**
*E*	**<0.001**	**<0.001**	0.466	**<0.001**	**<0.001**	**<0.001**	**<0.001**	**<0.001**	**<0.001**	**<0.001**
Φ_PSII_	**<0.001**	**0.001**	**<0.001**	**<0.001**	0.014	**<0.001**	**<0.001**	**<0.001**	**<0.001**	**<0.001**
Chl	**<0.001**	0.177	**<0.001**	**<0.001**	**0.003**	**0.009**	**<0.001**	**<0.001**	0.310	**0.003**
Flav	**<0.001**	0.077	**<0.001**	**<0.001**	0.180	**<0.001**	**<0.001**	**<0.001**	0.145	**0.036**
Anth	**<0.001**	**<0.004**	**<0.001**	**<0.001**	**<0.001**	**<0.001**	**<0.001**	0.697	0.126	0.387
AB	**<0.001**	**<0.001**	**<0.001**	**<0.001**	0.421	**<0.001**	**<0.001**	0.793	0.128	**<0.001**
BB	**<0.001**	**<0.001**	**<0.001**	**<0.001**	0.064	**<0.001**	0.070	**0.006**	0.070	**0.006**
R/S	**<0.001**	0.614	**<0.001**	**<0.001**	0.451	0.052	**<0.001**	0.051	0.824	0.640
C:N	0.057	**<0.001**	**<0.001**	**<0.001**	**<0.001**	**<0.001**	**0.015**	0.219	**0.040**	0.402

Factors: S, species; CO_2_, atmospheric CO_2_ concentration; WA, water availability; T, temperature. Measured physiological, morphological and biochemical parameters: RWC, relative water content; *A*_max_, light-saturated rate of CO_2_ assimilation; *G*_S_, stomatal conductance; *E*, transpiration rate; Φ_PSII_, actual quantum yield of photosystem II; Chl, chlorophyll index; Flav, flavonol index; Anth, anthocyanin index; AB, above-ground dry biomass per plant; BB, below-ground dry biomass per plant; R/S, root to shoot ratio (BB/AB); C:N, C:N ratio in plant dry matter. The *p*-values are shown. Statistically significant effects are in bold (*p* ≤ 0.05).

### 3.3 Effects on physiological traits

**Figure 4:**
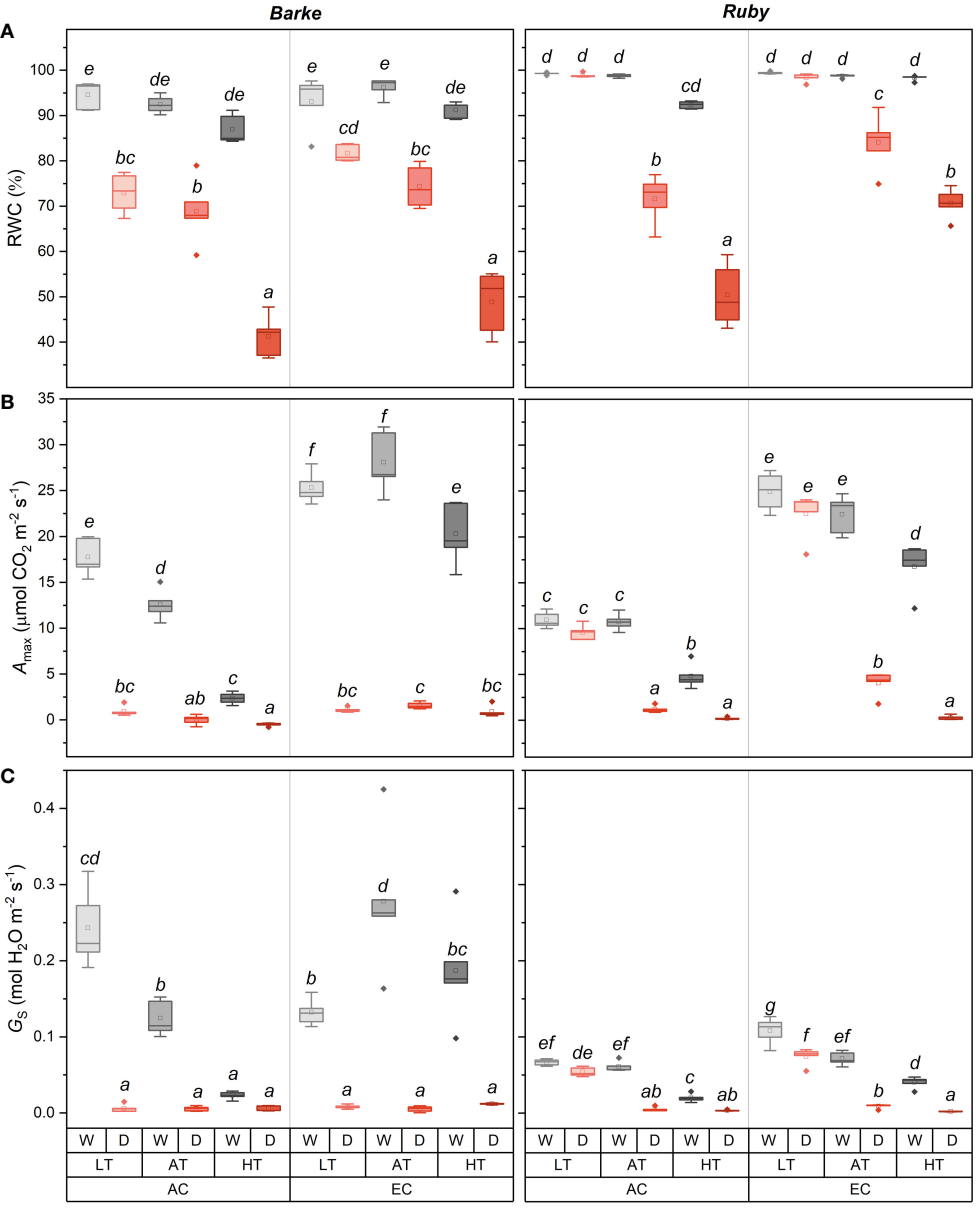
Box-plots showing the effect of CO_2_ concentration, temperature, and water availability on leaf relative water content (RWC, **(A)**), light-saturated CO_2_ assimilation rate (*A*_max_, **(B)**), and stomatal conductance (*G*_S_, **(C)**) for spring barley (Barke, left) and sorghum (Ruby, right) separately. AC, ambient CO_2_ concentration (400 ppm); EC, elevated CO_2_ concentration (800 ppm); LT, low temperature (21/7°C); AT, ambient temperature (26/12°C); HT, high temperature (33/19°C); W, well-watered control; D, drought stress. The lower and upper limits of the box represent the 25-75% percentile. The horizontal line inside the box represents the median and the point in the middle of the box the mean. Error bars represent the 1.5 interquartile range. The points outside the error bars represent outliers. Letters above the boxes represent homogeneous groups of *post-hoc* testing following a three-way ANOVA (separate CO_2_, temperature and drought effects for each species/genotype) using Tukey’s test at p=0.05 (variants with different letters within a species/genotype show a statistically significant difference between means).

Relative water content (RWC) was affected in both species mainly by drought, but specific interactions with temperature, CO_2_ concentration and species are evident (**Figure 4A**). In barley, drought induced a statistically significant decline in RWC in all temperature and CO_2_ concentration treatments. However, the magnitude of RWC reduction significantly increased with temperature, irrespective of CO_2_ concentration. EC in barley slightly alleviated the drought-induced decrease in RWC, but this effect was not statistically significant (*p* > 0.05). HT also reduced RWC in well-watered barley plants, but this effect was relatively small and statistically insignificant. Generally, drought and temperature-induced reduction of RWC was less pronounced in sorghum than in barley. This is particularly obvious at low temperatures (LT) where drought, irrespective of CO_2_ concentration, did not affect RWC in sorghum. Therefore, the statistically significant decrease in RWC due to drought was observed in sorghum only in AT and HT, and at the same time, there were also significant differences in the decrease of RWC between AT and HT treatments, with a greater decrease in RWC achieved in HT at both CO_2_ concentrations. In sorghum, EC mitigated the drought-induced decline in RWC in both AT and HT treatments.

The light-saturated rate of CO_2_ assimilation, *A*_max_ (**Figure 4B**), was mainly reduced by drought and increased by elevated CO_2_ concentration. In barley, the effect of drought was statistically significant in all three temperature treatments (LT, AT and HT) and both CO_2_ concentrations (AC and EC). The increase in *A*_max_ due to EC was statistically significant for both species in all well-watered treatments within all temperature treatments (LT, AT and HT). The effect of EC in drought-stressed plants was generally small but statistically significant under AT and HT treatments in barley and under LT and AT treatments in sorghum. In barley, a decrease in *A*_max_ due to HT was also evident in the well-watered treatments. The decline of *A*_max_ due to HT was more pronounced under AC as compared to EC conditions. Thus, EC in well-watered barley plants mitigated the negative effect of HT on *A*_max_. Sorghum showed significantly different responses compared to barley, especially in response to drought at LT treatment. The decrease in *A*_max_ due to drought was significantly lower in both CO_2_ concentrations (AC and EC). Under EC, the *A*_max_ decrease in sorghum due to drought was also lower in AT, while the *A*_max_ value under drought stress was significantly higher in AT compared to HT, unlike in barley. The relative effect of EC on *A*_max_ in well-watered control was also higher in sorghum, although there is no obvious interaction with temperature as shown in barley. At both CO_2_ concentrations, *A*_max_ decreased with increasing temperature in well-watered plants, which decrease was particularly obvious at HT conditions.

Stomatal conductance (*G*_S_) showed a similar response pattern as *A*_max_ with more pronounced species-specific differences which were particularly evident in well-watered plants (**Figure 4C**). Among others, well-watered sorghum plants had generally lower *G*_S_ values than barley, but these differences are negligible at HT and drought treatments. Compared to *A*_max_, *G*_S_ was also less affected by CO_2_ concentration. In barley, a statistically significant decrease in *G*_S_ due to drought was evident, except for the AC HT treatment. The increase in temperature significantly decreased *G*_S_ for the well-watered plants under AC, when comparing AT versus LT, HT versus AT, and HT versus LT. In contrast, *G*_S_ showed a different response to temperature under EC. There was a statistically significant increase in *G*_S_ in AT versus LT, but a decrease in *G*_S_ between AT and HT. However, HT vs. LT showed a statistically significant increase in EC. Thus, it was again evident that, in barley, the negative effect of high temperature on *G*_S_ was mitigated by elevated CO_2_ concentration under well-watered conditions. Besides generally lower *G*_S_ values in sorghum than in barley, a significantly lower effect of drought at LT was evident for both AC and EC conditions in sorghum. In both cases, stomata remained open under drought although the decrease in *G*_S_ was statistically significant under EC conditions. Increasing temperature led to a significant decrease in *G*_S_ of well-watered sorghum plants, which decline was particularly obvious under EC conditions. A similar response to experimental factors was also found for transpiration rate (*E*, **Supplementary Figure S1A**) with a generally less pronounced effect of temperature. The dominant effect of drought and the interaction of water availability and temperature were also observed for the actual quantum yield of photosystem II (Φ_PSII_, **Supplementary Figure S1B**). Drought in combination with AT and especially in combination with HT decreases Φ_PSII_. This effect was significantly higher in sorghum than barley and under AC than EC conditions.

### 3.4 Effects on above- and below-ground biomass

**Figure 5:**
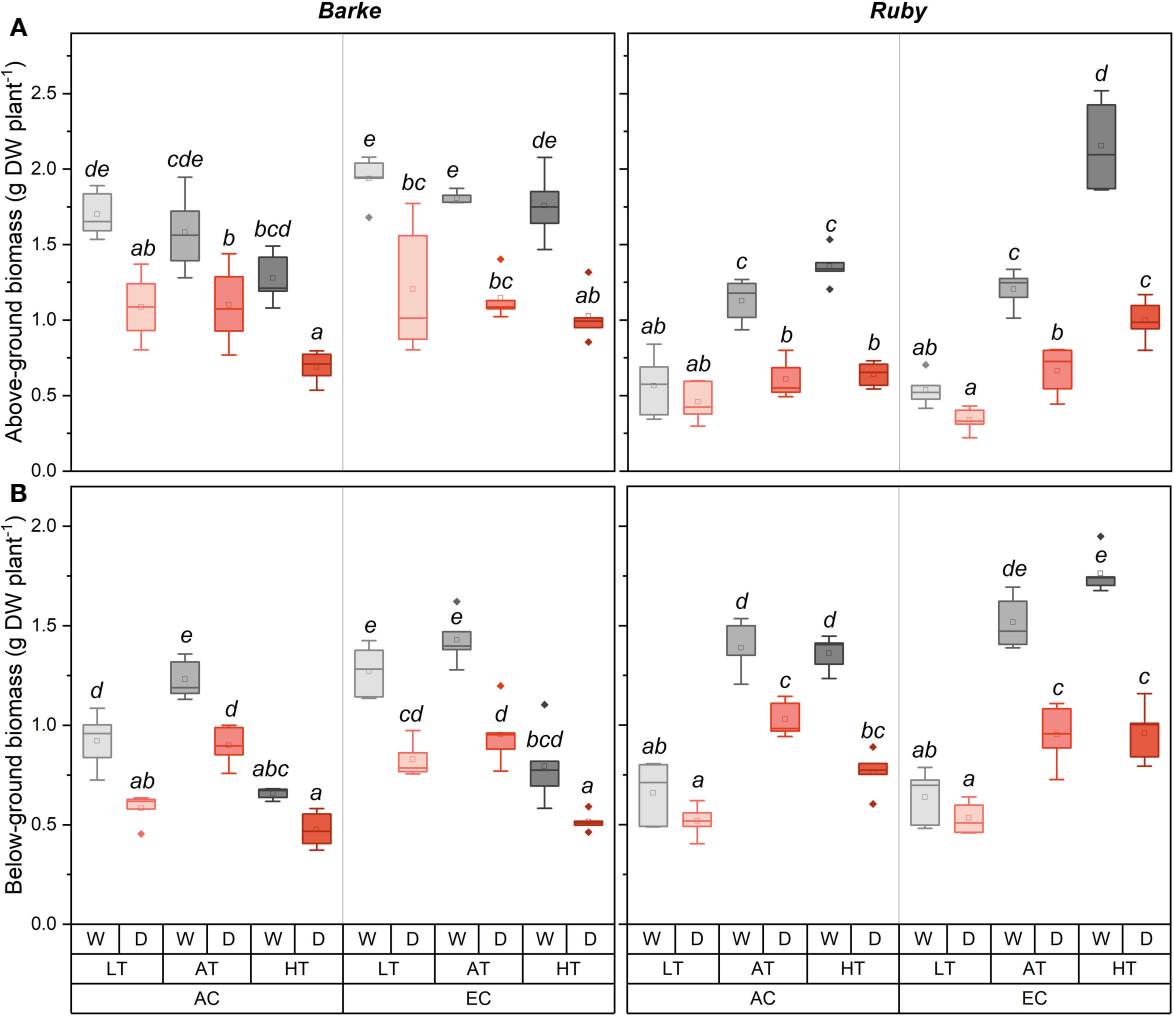
Box-plots showing the effect of CO_2_ concentration, temperature, and water availability on dry above-ground biomass **(A)** and dry below-ground biomass **(B)** per plant of spring barley (variety Barke, left) and sorghum (variety Ruby, right). AC, ambient CO_2_ concentration (400 ppm); EC, elevated CO_2_ concentration (800 ppm); LT, low temperature (21/7°C); AT, ambient temperature (26/12°C); HT, high temperature (33/19°C); W, well-watered control; D, drought stress. The lower and upper limits of the box represent the 25-75% percentile. The horizontal line inside the box represents the median and the point in the middle of the box the mean. Error bars represent the 1.5 interquartile range. The points outside the error bars represent outliers. Letters above the boxes represent homogeneous groups of *post-hoc* testing following a three-way ANOVA (separate CO_2_, temperature and drought effects for each species/genotype) using Tukey’s test at *p*=0.05 (variants with different letters within a species/variety show a statistically significant difference between means).

Above-ground biomass was generally higher in barley than in sorghum, but an opposite result was found under combined conditions of HT and EC (**Figure 5A**). Generally, above-ground biomass tended to decrease with increasing temperature in barley, while it proportionally increased in sorghum. However, the temperature-induced decrease in above-ground biomass of barley was statistically significant only under combined conditions of AC, HT and D. Under EC, the temperature effect on above-ground biomass was diminished. In well-watered sorghum plants, above-ground biomass significantly increased with increasing temperature regardless of CO_2_ concentration, except for AC AT and AC HT counterparts. Similar temperature-induced trends were also found in drought-treated sorghum plants. Generally, compared to well-watered plants, drought caused a decrease in above-ground biomass (**Figure 1B**). This decrease was statistically significant in barley across all temperature and CO_2_ treatments, however, the effect was slightly larger under EC conditions. For sorghum, the negative effect of drought on above-ground biomass increased with increasing temperature under both CO_2_ concentrations. The positive effect of EC on above-ground biomass was significantly pronounced under HT conditions in both well-watered and drought-stressed sorghum plants.

Below-ground biomass was dominantly affected by temperature, drought, and, to a lesser extent, by the interaction with CO_2_ concentration (**Figure 5B**). Although there were only relatively small differences in below-ground biomass between the two species, these were particularly evident under combined conditions of HT and EC where the two species showed different responses and higher values of below-ground biomass were shown in sorghum compared to barley. Drought generally caused a decrease in below-ground biomass. This decrease was statistically significant in barley for all combinations of CO_2_ concentration and temperature. In contrast, in sorghum, drought had no significant effect under LT, regardless of CO_2_ concentration. On the contrary, in AT and HT the effect of drought on below-ground biomass was statistically significant. In barley, below-ground biomass was highest in AT and towards LT and HT below-ground biomass decreased similarly in the well-watered and drought-stressed plants. Except for the comparison of LT vs. AT for EC variants, and LT vs. HT under drought stress for AC variants, the differences in below-ground biomass due to temperature were statistically significant in barley. While for barley the highest decrease in below-ground biomass was observed at HT, the opposite was observed for sorghum. EC increased below-ground biomass of barley statistically significantly only under LT, irrespective of water availability. In sorghum, the effect of EC was significant only in combination of HT with well-watered treatments.

Drought negatively affected slightly more above-ground biomass compared to below-ground biomass, which was evident in the increase of R/S ratio (**Supplementary Figure S2C**). Although the changes in R/S ratio due to drought were statistically significant only in sorghum for the combined treatment of AC and AT, it was generally evident that R/S ratio changed more in sorghum than barley. For both species, there was also observed a decrease in R/S ratio at HT and generally the highest values were observed at AT. When comparing the species, sorghum had a higher R/S ratio, practically twice as high as barley.

### 3.5 Effects on biochemical parameters

**Figure 6:**
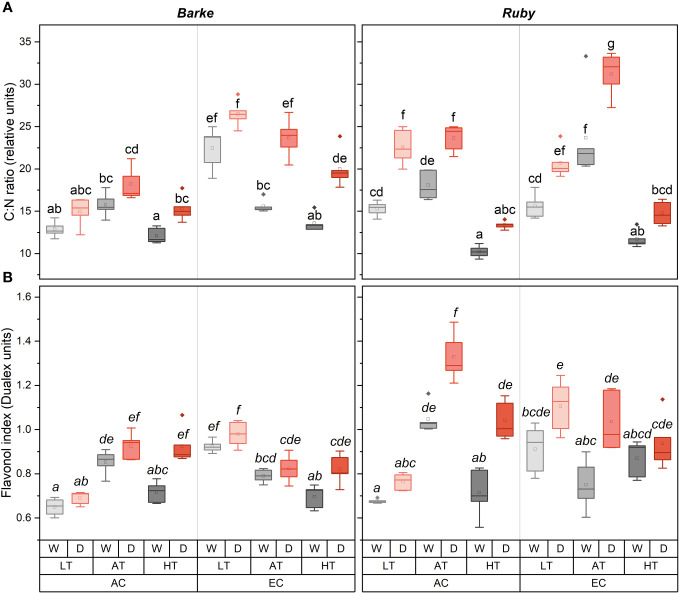
Box-plots showing the effect of CO_2_ concentration, temperature, and water availability on C:N ratio in the above-ground dry matter **(A)** and flavonol index **(B)** for spring barley (variety Barke, left) and sorghum (variety Ruby, right) separately. AC, ambient CO_2_ concentration (400 ppm); EC, elevated CO_2_ concentration (800 ppm); LT, low temperature (21/7°C); AT, ambient temperature (26/12°C); HT, high temperature (33/19°C); W, well-watered control; D, drought stress. The lower and upper limits of the box represent the 25-75% percentile. The horizontal line inside the box represents the median and the point in the middle of the box the mean. Error bars represent the 1.5 interquartile range. The points outside the error bars represent outliers. Letters above the boxes represent homogeneous groups of *post-hoc* testing following a three-way ANOVA (separate CO_2_, temperature and drought effects for each species/genotype) using Tukey’s test at *p*=0.05 (variants with different letters within a species/variety show a statistically significant difference between means).

The C:N ratio showed a very strong response to all experimental factors, including species effects (**Figure 6A**). In particular, the drought increased the values of the C:N ratio across all combinations of experimental factors. The effect of temperature on the C:N ratio has a typical non-linear character with a maximum under AT, while lower C:N values were achieved at HT and LT treatments. Only in EC barley plants, the highest values of the C:N ratio were reached at LT, and the C:N ratio gradually decreased towards higher temperatures. Under AT conditions, the highest C:N ratio was observed in sorghum plants grown under EC irrespective of water availability. The effect of EC on the C:N ratio shows interactions with species and temperature. While in sorghum the effect of EC on C:N ratio increase was statistically significant only under AT, in barley the significant increase of C:N ratio was found under LT for both water availability treatments, and in AT and HT only in drought-stressed plants.

Flav index showed higher values and a greater sensitivity to experimental factors in sorghum than in barley (**Figure 6B**). Drought generally increased Flav in both species. In barley, drought effects were statistically significant in HT plants under both CO_2_ treatments. In sorghum, the drought-induced increase in Flav was evident under AC for the AT and HT treatments, while under EC a significant increase was observed for AT treatment. The effect of temperature on Flav showed an interaction with CO_2_ concentration. The highest Flav was observed under AC in AT treatment for both barley and sorghum. In barley was this effect statistically significant for well-watered treatments in comparison to both LT and HT, while for drought-stressed variants the difference was significant only in comparison to LT. In sorghum this effect was significant in comparison to both LT and HT irrespective of water availability. Within the EC treatments, Flav showed a slight decrease due to increasing temperature, however, this effect was statistically significant only in barley for comparisons between LT and AT, or between LT and HT. The effect of EC on Flav showed an interaction mainly with temperature, with the significant increase in both barley and sorghum under LT and in sorghum with a significant decrease under AT, irrespective of water availability treatment.

In contrast to Flav, Anth index reached higher values in barley, while it was generally very low in sorghum (**Supplementary Figure S2B**). Temperature sensitivity of Anth also differed between species. While the highest values were reached at lower temperatures in barley, HT treatment, particularly in combination with drought, resulted in a substantial increase of Anth in sorghum.

Chl index showed generally higher values in barley compared to sorghum (**Supplementary Figure S2A**). The effect of drought on Chl was only evident in barley under LT regardless of CO_2_ concentration and further under AT in combination with EC. In barley, the highest Chl values were generally achieved under AT, while sorghum showed an increase with temperature up to HT (for AC treatments) or very small changes with temperature for EC treatments.

## 4 Discussion

The changes in weather extremes have already led to a tripling of crop yield losses over the last fifty years (Brás et al., 2021). However, the resulting yield impacts are largely modulated by the interactions of various environmental drivers which can range from synergistic to antagonistic (Mittler, 2006). Some authors suggest that the negative effect of reduced water availability will be largely offset by the positive effect of elevated CO_2_ concentrations (Swann et al., 2016). In contrast, Gray et al. (2016) expect a rather higher likelihood of a negative interaction based on experimental results. To understand these conflicting results and the conditions under which a given type of response occurs, it is necessary to analyse the responses under the conditions of the so-called multifactorial stress combination (Zandalinas and Mittler, 2022), which was the aim of this study along with understanding the differences in response to multifactorial stress between C3 and C4 crops.

As shown by Cohen et al. (2021) the type of interaction effects of heat and drought stress are largely dependent on the photosynthetic metabolism of the crop, with C3 crops responding with higher yield loss to heat stress compared to C4 plants, while the opposite is true for drought stress. However, the combined effect of heat and drought stress on yield does not differ much between C3 and C4 crops. Higher sensitivity of C4 crops to drought stress was also documented by Bellasio et al. (2023) despite higher water use efficiency in C4 crops. Even more complex are the interactions between elevated CO_2_ concentration and other abiotic environmental factors in relation to the type of photosynthetic metabolism. Vijayalakshmi et al. (2023) showed that elevated CO_2_ concentration is better able to mitigate the negative effects of drought and heat stress on physiology and yield in C3 crops compared to C4 crops.

Our study proves that both species show a similar response of physiological parameters to temperature under sufficient water supply. Such a result indicated a general decrease in photosynthetic performance at HT in both C3 and C4 species. In contrast, sorghum showed the opposite response in production parameters, indicating an increase in biomass up to the highest temperatures. These differences imply a decoupling of the physiological response from the growth response in sorghum and allowing thus to compensate for the negative physiological response to temperature and achieve high biomass production. This may be due to the significantly higher enzymatic and non-enzymatic antioxidant activity of C4 plants, which protects against oxidative damage (Stepien and Klobus, 2005; Nayyar and Gupta, 2006) at least for the first part of stress exposure. The higher non-enzymatic antioxidant capacity in this study was indicated by the higher Flav values in sorghum compared to barley, which also showed a higher response (increase) to drought in sorghum. As the PCA analysis demonstrated, there was a negative association between the effect of HT and the effect of EC, however, this effect was significantly greater in barley. This effect was particularly evident in the alleviation of the negative impact of high temperature on the CO_2_ assimilation rate (*A*_max_). In sorghum, on the other hand, the interaction between HT and EC was particularly evident for above-ground biomass, with an amplification of the positive effect. In contrast, Hamilton et al. (2008) reported an inverse interaction between temperature and EC on CO_2_ assimilation rate, i.e. a moderating effect in C3 and an enhancing effect in C4 plants. Similarly, Wang et al. (2008) documented an increased thermotolerance of CO_2_ assimilation rate in C3 plants and conversely a decreased thermotolerance in C4 plants. C3 plants generally acclimate to high temperatures by a decrease in respiration rate, increased electron transport capacity and synthesis of Rubisco activase with high heat stability. In addition, significant shifts in temperature optima of photosynthesis were reported both in C3 and C4 plants grown under elevated CO_2_ concentration (Sage and Kubien, 2007). Generally, the temperature optima for photosynthesis of C4 plants are higher than those of C3 plants, but their range is relatively narrow (Yamori et al., 2014). This means that any change in temperature from the optimum induces a more pronounced decline in CO_2_ assimilation rate in C4 plants than in C3 plants, and that C4 plants have relatively higher photosynthetic efficiency at HT compared to C3 plants. This was rather confirmed by our results on above- and below-ground biomass production, while *A*_max_ showed even higher sensitivity to HT in sorghum.

The effect of drought on both physiological and biomass production parameters was significantly modulated by temperature in sorghum, but not in barley. Drought also did not decouple the rate of *A*_max_ from above- and below-ground biomass formation. The interaction of drought with LT in sorghum was weak but increased with increasing temperature. C4 plants are able to achieve the same rate of CO_2_ assimilation at significantly lower stomatal conductance than C3 plants leading to high water use efficiency, particularly at lower temperatures (Killi et al., 2017). Accordingly, differences in water use efficiency between C4 and C3 plants are substantial at low temperatures but decline at high temperatures (Killi et al., 2017). This was confirmed by physiological parameters, including RWC, in our study. The negative effect of drought on RWC was also mitigated by EC in sorghum, whereas this effect was negligible in barley. Similar effects of EC and drought on RWC were reported in tall fescue by Yu et al. (2012). However, the effect of elevated CO_2_ concentration on RWC has been reported to be species-specific (Miranda-Apodaca et al., 2018). We found that sorghum was able to maintain higher RWC values than barley even at HT and drought (especially under EC conditions). The relative impact of drought increased significantly with increasing temperature in sorghum, whereas it remained at a similar level in barley. This was probably due to the fact that C4 plants respond physiologically to a decrease in water potential much earlier and thus show a higher sensitivity to water loss (Bellasio et al., 2023), although they exhibit lower water losses. EC shifts the threshold of water availability for a decrease in CO_2_ assimilation rate to lower values (resulting in higher drought tolerance) more in C3 as compared to C4 plants (Cao et al., 2022). At EC, the stomatal response of C3 and C4 plants to drought is thus approximated, but due to the significantly higher non-stomatal sensitivity of C4 plants, the performance of C4 plants under conditions of reduced water availability and EC is actually impaired (Bellasio et al., 2018).

Our results show a significantly higher R/S ratio in C4 sorghum and also the ability to increase this ratio under drought conditions. This adaptive potential of sorghum can, under storage-driven environments (Bodner et al., 2015), mean better performance in future climates. In particular, an improved ability to acquire the water reserves in the soil, especially from deeper soil layers, can be expected. The possible reason for stimulated root growth and reduced shoot growth under drought could be to gain access to water and minimise water loss, respectively (Gupta et al., 2020).

The C:N ratio can generally be considered as an indicator of the triggering defence mechanisms in plants, which responds to environmental stimuli and leads to morphological and biochemical adjustments enabling them to overcome stress (Klem et al., 2019; Klem et al., 2022). In the present study, the C:N ratio was positively influenced mainly by drought and EC, while it was reduced by HT. The interplay between N and C metabolism is modulated by the expression of phenylpropanoid-flavonoid biosynthetic genes. This results in an increased accumulation of flavonoids (Olsen et al., 2009), which was also corroborated in the current study. The C:N ratio also showed positive correlations in both species to another adaptive trait, R/S ratio, and in the case of sorghum also to accumulation of anthocyanins (Anth).

Considering that C4 crops show a greater decrease in production due to higher temperature and drought in the experimental results so far, and the mitigating effect of EC is practically absent, a higher climate change related yield decline can generally be expected for C4 as compared to C3 crops (Makowski et al., 2020). Our results generally confirm this except for the combination of HT with EC and sufficient water availability, where C4 sorghum is able to outperform barley in biomass production, especially relative to AC and LT conditions. The lower water loss, higher RWC and marginal decrease in CO_2_ assimilation rate due to drought at LT in sorghum do not reflect positively on sorghum biomass production. This is due to the fact that sorghum generally has low biomass production at LT.

## 5 Conclusions

The results of the present study demonstrate the decoupling of photosynthetic CO_2_ assimilation and production parameters in C4 sorghum grown under HT (33/19°C) and/or combined conditions of HT and EC. Despite the higher sensitivity of C4 photosynthesis to HT, it significantly increases above- and below-ground biomass of sorghum under sufficient water availability and EC (800 ppm CO_2_) conditions, which indicates the role of higher antioxidative capacity or other defence mechanisms at least for the first part of heat stress. In contrast, the negative effect of drought on biomass production is amplified by increasing temperature, even though drought induces some acclimation mechanisms in sorghum, such as an increase in the C:N ratio and the associated increases in Flav or R/S ratio. These complex interactions of individual factors imply that, unlike C3 barley, C4 sorghum can have a much higher variability in responses to changing environmental factors and thus a more difficult prediction of climate change impacts. Nevertheless, under certain conditions (combination of EC, HT and sufficient water availability) the outperforming of C4 crops can be expected. C3 crops are expected to perform better under conditions of lower temperatures, even when combined with drought. However, under insufficient water availability (storage-driven environment), the negative response of C4 sorghum can be compensated by an adjusted R/S ratio and increased accumulation of antioxidants. The significant interactions between the type of photosynthetic metabolism and temperature and between the effect of temperature and water availability indicate that future research on refining the production potential of C3 and C4 crops under expected climate change should focus primarily on these factors and their combinations. It can be assumed that the strong effect of these factors can significantly overwhelm the positive effect of elevated CO_2_ concentration, which is still rather overestimated in the models. It will also be important for future research to better understand the importance of high temperature and drought in C4 crops, given that they show a positive effect of temperature but a strong antagonistic response to its combination with drought and therefore may only be suitable for certain environments. Acquiring this knowledge is a key prerequisite for upscaling to larger areas and assessing the potential for changes in the proportion of C3 and C4 crops using updated growth models.

## Data availability statement

The original contributions presented in the study are included in the article/**Supplementary Material**. Further inquiries can be directed to the corresponding author.

## Author contributions

EO: Data curation, Investigation, Methodology, Writing – original draft, Writing – review & editing. PPS: Conceptualization, Formal analysis, Investigation, Methodology, Visualization, Writing – original draft, Writing – review & editing. HF: Formal analysis, Investigation, Methodology, Writing – review & editing. PH: Formal analysis, Writing – review & editing, Methodology. OU: Conceptualization, Formal analysis, Writing – review & editing. KK: Conceptualization, Formal analysis, Funding acquisition, Supervision, Writing – review & editing.

## Supplementary material

Supplementary Figure 1 Box-plots showing the effect of CO_2_ concentration, temperature, and water availability on the transpiration rate (*E*, **(A)**) and actual quantum yield of photosystem II (Φ_PSII_, **(B)**) for spring barley (variety Barke, left) and sorghum (variety Ruby, right) separately. AC, ambient CO_2_ concentration (400 ppm); EC, elevated CO_2_ concentration (800 ppm); LT, low temperature (21/7°C); AT, ambient temperature (26/12°C); HT, high temperature (33/19°C); W, well-watered control; D, drought stress. The lower and upper limits of the box represent the 25-75% percentile. The horizontal line inside the box represents the median and the point in the middle of the box the mean. Error bars represent the 1.5 interquartile range. The points outside the error bars represent outliers. Letters above the boxes represent homogeneous groups of *post-hoc* testing following a three-way ANOVA (separate CO_2_, temperature and drought effects for each species/genotype) using Tukey’s test at *p*=0.05 (variants with different letters within a species/genotype show a statistically significant difference between means).

Supplementary Figure 2 Box-plots showing the effect of CO_2_ concentration, temperature, and water availability on the chlorophyll index **(A)**, anthocyanin index **(B)** and root-to-shoot ratio **(C)** for spring barley (Barke, left) and sorghum (Ruby, right) separately. AC, ambient CO_2_ concentration (400 ppm); EC, elevated CO_2_ concentration (800 ppm); LT, low temperature (21/7°C); AT, ambient temperature (26/12°C); HT, high temperature (33/19°C); W, well-watered control; D, drought stress. The lower and upper limits of the box represent the 25-75% percentile. The horizontal line inside the box represents the median and the point in the middle of the box the mean. Error bars represent the 1.5 interquartile range. The points outside the error bars represent outliers. Letters above the boxes represent homogeneous groups of *post-hoc* testing following a three-way ANOVA (separate CO_2_, temperature and drought effects for each species/genotype) using Tukey’s test at *p*=0.05 (variants with different letters within a species/genotype show a statistically significant difference between means).
