# Supplementary figures and images for: Differential physiological and production responses of C3 and C4 crops to climate factor interactions
Source: Front Plant Sci. 2024 Feb 2;15:1345462. doi: 10.3389/fpls.2024.1345462 (PMC10869619; doi:10.3389/fpls.2024.1345462)

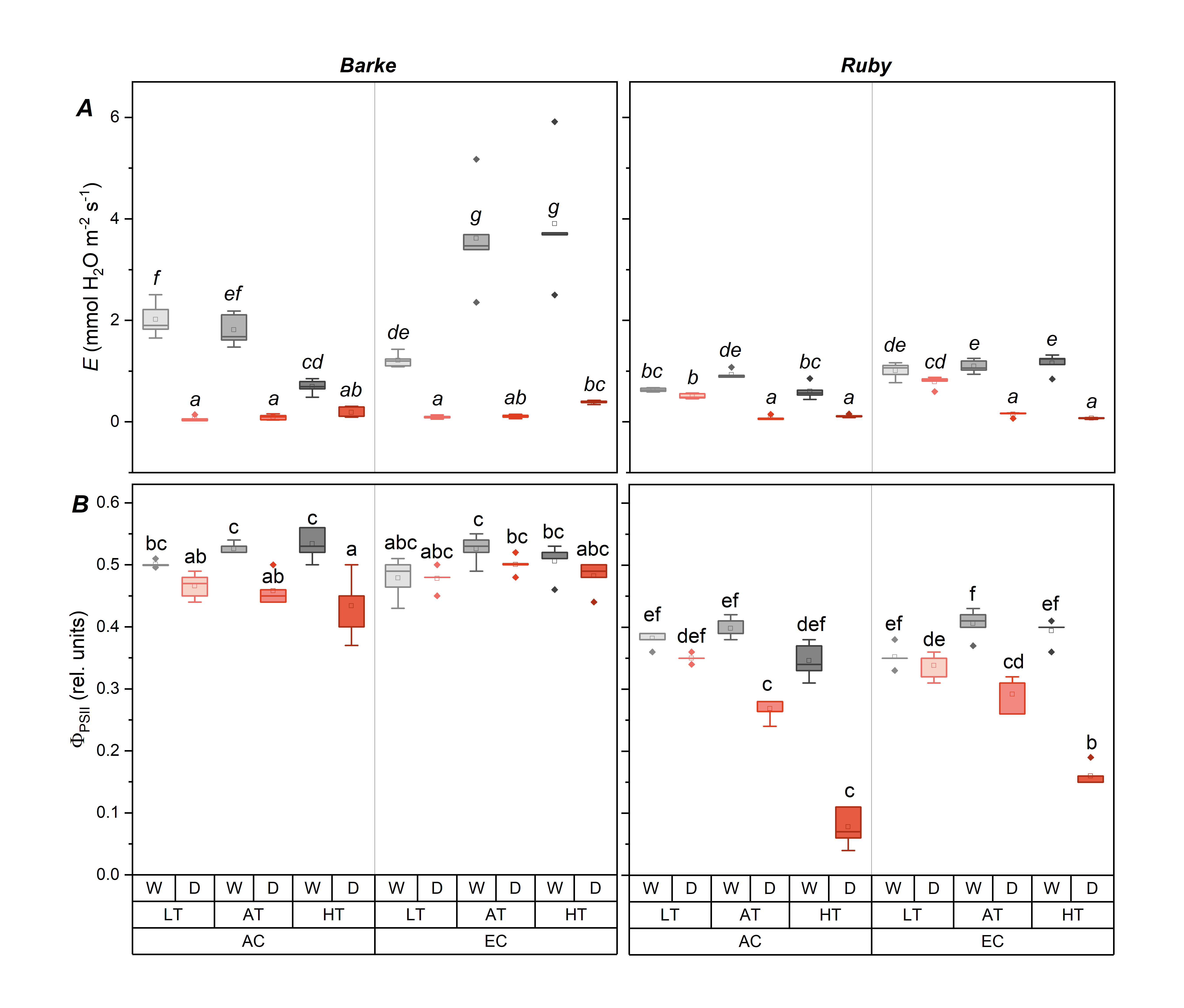

Supplement: Supplementary Figure 1 — Box-plots showing the effect of CO2 concentration, temperature, and water availability on the transpiration rate (E, (A)) and actual quantum yield of photosystem II (ΦPSII, (B)) for spring barley (variety Barke, left) and sorghum (variety Ruby, right) separately. AC, ambient CO2 concentration (400 ppm); EC, elevated CO2 concentration (800 ppm); LT, low temperature (21/7°C); AT, ambient temperature (26/12°C); HT, high temperature (33/19°C); W, well-watered control; D, drought stress. The lower and upper limits of the box represent the 25-75% percentile. The horizontal line inside the box represents the median and the point in the middle of the box the mean. Error bars represent the 1.5 interquartile range. The points outside the error bars represent outliers. Letters above the boxes represent homogeneous groups of post-hoc testing following a three-way ANOVA (separate CO2, temperature and drought effects for each species/genotype) using Tukey’s test at p=0.05 (variants with different letters within a species/genotype show a statistically significant difference between means). [file Image_1.jpg]

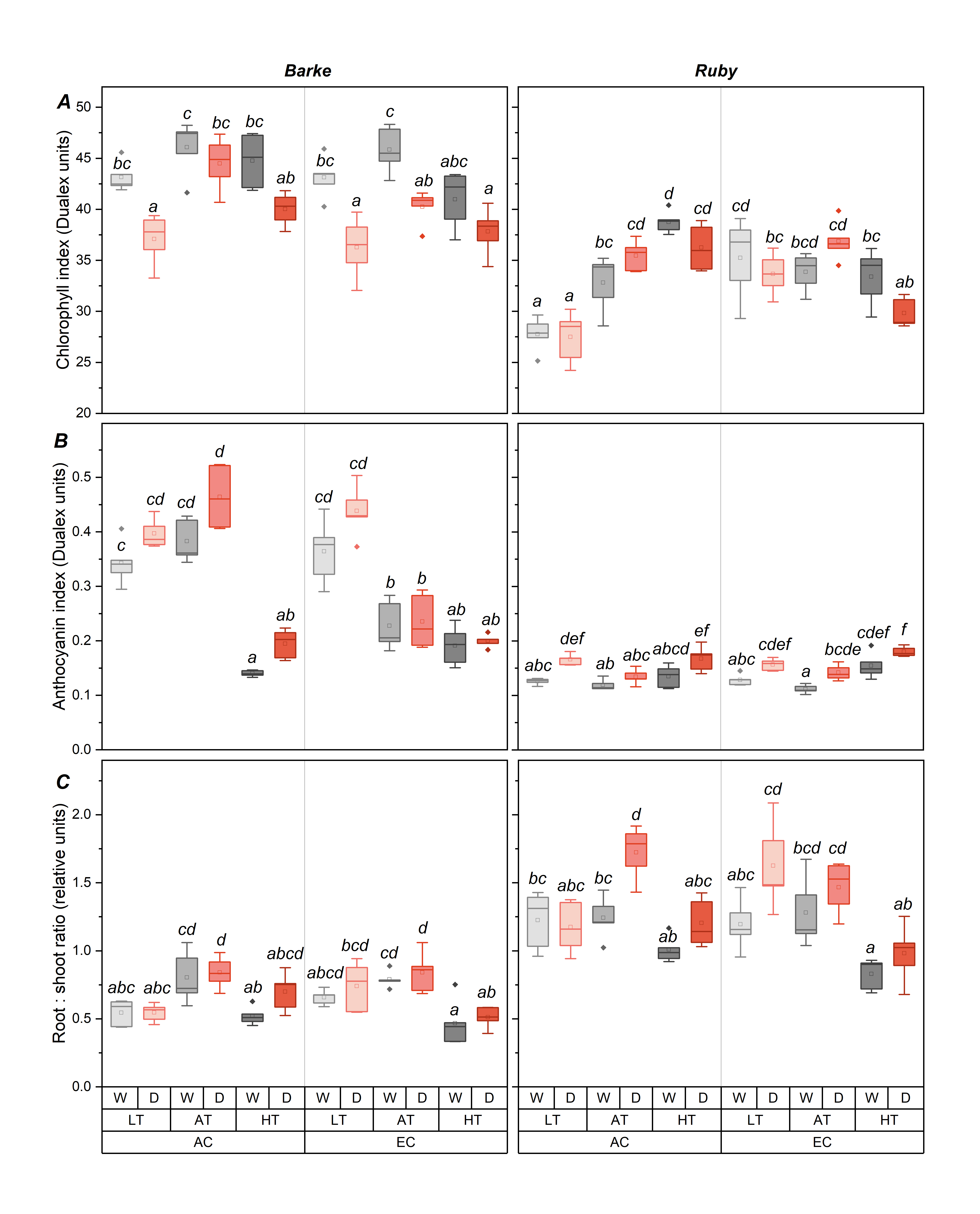

Supplement: Supplementary Figure 2 — Box-plots showing the effect of CO2 concentration, temperature, and water availability on the chlorophyll index (A), anthocyanin index (B) and root-to-shoot ratio (C) for spring barley (Barke, left) and sorghum (Ruby, right) separately. AC, ambient CO2 concentration (400 ppm); EC, elevated CO2 concentration (800 ppm); LT, low temperature (21/7°C); AT, ambient temperature (26/12°C); HT, high temperature (33/19°C); W, well-watered control; D, drought stress. The lower and upper limits of the box represent the 25-75% percentile. The horizontal line inside the box represents the median and the point in the middle of the box the mean. Error bars represent the 1.5 interquartile range. The points outside the error bars represent outliers. Letters above the boxes represent homogeneous groups of post-hoc testing following a three-way ANOVA (separate CO2, temperature and drought effects for each species/genotype) using Tukey’s test at p=0.05 (variants with different letters within a species/genotype show a statistically significant difference between means). [file Image_2.jpg]
